# Association of simple renal cysts with metabolic syndrome in adults

**DOI:** 10.3389/fpubh.2022.951638

**Published:** 2022-11-03

**Authors:** Wei-Chen Shen, Zih-Jie Sun, Chieh-Ying Chou, Yu-Tsung Chou, Feng-Hwa Lu, Yi-Ching Yang, Chih-Jen Chang, Jin-Shang Wu

**Affiliations:** ^1^Department of Family Medicine, National Cheng Kung University Hospital, College of Medicine, National Cheng Kung University, Tainan, Taiwan; ^2^Division of Family Medicine, National Cheng Kung University Hospital Dou-Liou Branch, College of Medicine, National Cheng Kung University, Yunlin, Taiwan; ^3^Department of Health Management Center, National Cheng Kung University Hospital, College of Medicine, National Cheng Kung University, Tainan, Taiwan; ^4^Department of Family Medicine, College of Medicine, National Cheng Kung University, Tainan, Taiwan; ^5^Department of Family Medicine, Ditmanson Medical Foundation Chia-Yi Christian Hospital, Chia-Yi, Taiwan

**Keywords:** renal cyst, metabolic syndrome, insulin resistance, hypertension, metabolic disease

## Abstract

**Background and aims:**

Metabolic syndrome is common nowadays and may increase risk of hypertension, type 2 diabetes mellitus, cardiovascular complications and even mortality. Renal cysts are also frequently found during routine examination. However, the relationship between simple renal cysts (SRCs) and metabolic syndrome remains unclear. This study aimed to investigate the association of SRCs with metabolic syndrome.

**Methods:**

A total of 16,216 subjects aged ≥18 years were enrolled in this study. SRCs were diagnosed with ultrasonography by finding: sharp, thin posterior walls, a round/oval shape, absence of internal echoes, and posterior enhancement. SRCs were categorized by number (0, 1, and ≥2) and size (<2 and ≥2 cm). Metabolic syndrome was diagnosed according to the consensus statement from the International Diabetes Federation.

**Results:**

In multivariate analysis, SRCs were positively related to metabolic syndrome (OR: 1.18, 95% CI: 1.06–1.34). The risk of metabolic syndrome was higher for SRCs with a number ≥2 (OR: 1.35, 95% CI: 1.08–1.68) and size ≥2 cm (OR: 1.33, 95% CI: 1.10–1.61). When considering the SRC number and size concomitantly, SRCs with a number ≥2/size ≥2 cm (OR: 1.42, 95% CI: 1.02–1.98) or <2/size ≥2 cm (OR: 1.30, 95% CI: 1.04–1.62) were positively related to metabolic syndrome.

**Conclusions:**

Simple renal cysts were found to be related to a higher risk of metabolic syndrome, and the association is more significant in those with larger (sizes ≥2cm) or plural (numbers ≥2) SRCs.

## Introduction

Renal cystic diseases are classified into inherited, developmental and acquired condition according to their etiologies (1, 2). Simple renal cysts (SRCs) are a common type of acquired cysts, or part of acquired cystic kidney diseases (3), and are often found incidentally. The prevalence of SRCs varies from 5.0 to 20.8% in different countries (4, 5). One cross-sectional study demonstrated that the prevalence was 10.7% among Taiwanese people (6), and it is believed that the incidence of SRCs increases with age (4, 7–9). SRCs are thought to be benign, asymptomatic lesions in clinical practice, rarely requiring invasive intervention (3). However, studies have revealed that SRCs were associated with hypertension, elevated serum creatinine, and hyperuricemia (4, 8–11). Thus, more studies are needed to elucidate whether SRCs are potentially related to other negative health impacts.

Although the definition of metabolic syndrome differs among organizations, the key components include insulin resistance, glucose intolerance, central obesity, hypertension, and dyslipidemia (12). The diagnostic criteria of metabolic syndrome are easily accessed and were applied rapidly in clinical practice (12–14). The importance of metabolic syndrome has been raised nowadays since the presence of metabolic syndrome increases the risk of hypertension, type 2 diabetes mellitus (DM), non-alcoholic fatty liver diseases (15), cardiovascular complications and mortality (16).

Previous studies have targeted the association of SRCs with individual cardio-metabolic related disorders, such as obesity (11) hypertension (10), prehypertension (10), renal dysfunction (7, 17), and hyperuricemia (18). However, study that focus on the relationship between metabolic syndrome and SRCs is lacking. Furthermore, whether the characteristics of SRCs, such as the size or number of SRCs have impacts on metabolic syndrome remains indeterminate. Considering that metabolic syndrome significantly raises the risk of DM, heart disease, and mortality, investigating the possible impact of SRCs on metabolic syndrome may provide one additional route for further researches and clinical practice of metabolic abnormalities. Thus, the aim of the present study was to clarify the association of SRCs with metabolic syndrome.

## Methods

### Study population

Subjects who underwent a health examination were recruited at the National Cheng Kung University Hospital from June 2001 to August 2009. A total of 16,216 subjects were enrolled for final analysis after excluding subjects aged <18 years, taking medication for obesity management, history of bariatric surgery, pregnant women, or history of or abdominal sonography with the following findings: renal stone, renal cyst with calcification, any cause of hydronephrosis, medullary sponge kidney disease, medullary cystic kidney disease, renal ectopia, polycystic kidney disease, renal tumor, renal transplantation and status post-nephrectomy, and incomplete data. This study was approved by the Institutional Review Board of National Cheng Kung University Hospital in Taiwan (IRB number: B-ER106-066).

A standardized self-reported questionnaire was completed by every participant and was used to obtain demographic information, medical history, and lifestyle habits, including smoking, alcohol drinking, and regular exercise. Current alcohol drinkers were defined as those who had at least one alcoholic drink per week for at least the previous 6 months (19). Current smoking status was defined as at least 20 cigarettes per month during the past 6 months. Regular exercise was defined as exercise at least three times per week for a minimum of 20 min (19).

Body height and weight were measured by a certified anthropometry instrument (to the nearest 0.1 cm and 0.1 kg, respectively). Body mass index (BMI) was calculated as weight (kilograms) divided by height (m) squared (kg/m^2^). Subjects with a BMI ≥27 kg/m^2^ were defined as obese according to the guidelines of the Department of Health in Taiwan (20). Waist circumference (WC) was measured from the midway between the lower rib margin and the iliac crest with a standing posture (to the nearest 0.1 cm) at the end of a normal expiration. After 5 min of rest, the right branchial artery blood pressure was measured in the supine position by using a DINAMAP vital sign monitor (model 1846SX; Critikon, Irvine, CA) with an appropriate-sized cuff. Two blood pressure readings were taken separately under an interval of at least 5 min. Hypertension was diagnosed by a positive history of hypertension and the reading from the right brachial artery showing a systolic blood pressure (SBP) of 140 mmHg or more, or diastolic blood pressure (DBP) 90 mmHg or more (21).

After overnight fasting for 12 h, all participants received blood sampling for biochemical examinations, which included the following: fasting plasma glucose (FPG), glycated hemoglobin (HbA1c), total cholesterol (TC), triglyceride (TG), high-density lipoprotein cholesterol (HDL-C), uric acid and creatinine. Serum TC, TG and HDL-C levels were determined in the central laboratory of NCKUH with an autoanalyzer (Hitachi 747E, Tokyo, Japan). Patients' 2-h post-load glucose (2-h PG) was checked after a 75-g oral glucose tolerance test for all participants without DM or pregnancy. DM was defined as a positive history, FPG ≥126 mg/dl, 2-h PG ≥200 mg/dl or HbA1c ≥6.5% (21).

All participants were divided into two groups by the presence of metabolic syndrome or not, which was defined according to the consensus statement from the International Diabetes Federation (22). Metabolic syndrome was diagnosed if a participant had central obesity (WC ≥90 cm in men or ≥80 cm in women) and fulfilled ≥2 of the following four components: (1) FPG ≥ 100 mg/dl or previously diagnosed type 2 diabetes; (2) BP ≥ 130/85 mmHg or under treatment of previously diagnosed hypertension; (3) HDL-C < 40 mg/dl in men or <50 mg/dl in women or under drug treatment for reduced HDL-C; and, (4) TG ≥ 150 mg/dl or under drug treatment for dyslipidemia. All participants underwent abdominal sonographic examination (Toshiba Xario/SSA = 660A Ultrasound Machine; Toshiba, Tokyo, Japan, with a 3.5-MHz transducer). The diagnosis of SRCs was based on the criteria of abdominal sonography, including: (1) sharp, thin posterior walls, (2) round/oval shape, (3) absence of internal echoes, and (4) posterior enhancement (23). We further categorized SRCs according to their sizes (<2 and ≥2 cm) and numbers (<2 and ≥2).

### Statistical analyses

Statistical analyses were performed by using Windows SPSS 17.0 statistical software (Chicago, Illinois, USA). In univariate analysis, the comparison of continuous variables was carried out using Student's *t*-tests and categorical variables by Pearson Chi square tests between two groups. A binary logistic regression model was performed to investigate whether the SRCs were associated with an increased risk of metabolic syndrome. Other independent factors included age, gender, obesity, creatinine, uric acid, alcohol drinking, smoking, and regular exercise. Furthermore, the associations of the size, number, and the combined characteristics of SRCs with metabolic syndrome were also examined. The *p*-value < 0.05 was considered as statistically significant.

## Results

A total of 16,216 subjects were enrolled and divided into two groups according to the presence of metabolic syndrome. Table 1 shows the clinical characteristic between participants with (*n* = 4,071) and without metabolic syndrome (*n* = 12,145). Subjects with metabolic syndrome were predominantly older males and tended to have higher BMI, SBP, DBP, FPG, cholesterol, TG, uric acid, and creatinine, as well as the prevalence of obesity, hypertension, DM, and current tobacco and alcohol consumption, together with a lower HDL-C and prevalence of regular exercise.

**Table 1:** Comparisons of clinical characteristics between subjects with and without metabolic syndrome.

**Variables**	**Metabolic syndrome**		***p*-Value**
	**No (*n* = 12,145)**	**Yes (*n* = 4,071)**	
Age, years	47.6 ± 12.6	54.3 ± 12.0	<0.001
Male	6,959 (57.3)	2,751 (67.6)	<0.001
BMI, kg/m^2^	23.5 ± 3.2	27.1 ± 3.3	<0.001
BMI ≥27 kg/m^2^	1,440 (42.6)	1,941 (47.7)	<0.001
Waist circumference, cm	81.0 ± 12.0	115.6 ± 1,457.9	0.009
Central obesity	2,866 (23.6)	3,453 (84.8)	<0.001
SBP, mmHg	114.8 ± 15.8	131.4 ± 18.6	<0.001
DBP, mmHg	67.9 ± 10.1	76.9 ± 11.1	<0.001
Elevated BP^*^	1,867 (15.4)	2,319 (57)	<0.001
Hypertension	1,357 (11.2)	1,843 (45.3)	<0.001
FPG, mg/dl	89.3 ± 17.0	111.2 ± 40.1	<0.001
Hemoglobin A1c, %	5.6 ± 0.7	6.4 ± 1.5	<0.001
Hyperglycemia (FPG >100 mg/dl)	1,103 (9.1)	2,274 (55.9)	<0.001
Diabetes mellitus	1,128 (9.3)	1,662 (40.8)	<0.001
Cholesterol, mg/dl	194.5 ± 36.7	204.0 ± 39.2	<0.001
Triglyceride, mg/dl	108.5 ± 61.8	207.5 ± 133.1	<0.001
Hypertriglyceridemia (TG >150 mg/dl)	1,746 (14.4)	2,886 (70.9)	<0.001
HDL-C, mg/dl	52.0 ± 13.5	39.1 ± 9.3	<0.001
Low HDL-C^**^	3,023 (24.9)	3,092 (76)	<0.001
Creatinine, mg/dl	0.87 ± 0.40	0.93 ± 0.44	<0.001
eGFR, ml/min/1.73m^2^	95.2 ± 16.0	88.3 ± 16.9	<0.001
Uric acid, mg/dl	5.9 ± 1.5	6.7 ± 1.6	<0.001
ALT, U/L	29.7 ± 32.8	43.6 ± 40.3	<0.001
Current alcohol consumption	1,344 (11.1)	525 (12.9)	0.002
Current smoking	1,234 (10.2)	556 (13.7)	<0.001
Regular exercise ≥3/week	1,010 (8.3)	279 (6.9)	0.003

Data expressed as means ± standard deviation or number (percent).

BMI, body mass index; WC, waist circumference; SBP, systolic blood pressure; DBP, diastolic blood pressure; FPG, fasting plasma glucose; ALT, alanine aminotransferase; HDL-C, high-density lipoprotein-cholesterol; eGFR, estimated glomerular filtration rate.

* Elevated BP: SBP ≥130 mmHg or DBP ≥85 mmHg.

** Low HDL: HDL < 40 mg/dl in males and < 50 in females.

Table 2 further demonstrated that subjects with SRCs have higher prevalence of metabolic syndrome. It also showed that those with larger (size ≥2 cm) or plural (number ≥2) SRCs were more likely to have metabolic syndrome.

**Table 2:** Relationship between metabolic syndrome and renal cyst by Pearson Chi-square test.

**Variables**	**Metabolic syndrome**		***p*-Value**
	**No (*n* = 12,145)**	**Yes (*n* = 4,071)**	
**Presence of renal cyst**			
No	11,026 (90.8)	3,487 (85.7)	<0.001
Yes	1,119 (9.2)	584 (14.3)	
**Renal cyst, number**			
< 2	854 (7.0)	409 (10.0)	<0.001
≥2	265 (2.2)	32 (4.3)	
**Renal cyst, size**			
< 2 cm	747 (6.2)	347 (8.5)	<0.001
≥2 cm	372 (3.1)	237 (5.8)	
**Renal cyst, number and size**			
Number < 2 and size < 2 cm	588 (4.8)	246 (6.0)	<0.001
Number ≥2 and size < 2 cm	159 (1.3)	101 (2.5)	
Number < 2 and size ≥2 cm	266 (2.2)	163 (4.0)	
Number ≥2 and size ≥2cm	106 (0.9)	74 (1.8)	

Data expressed as means ± standard deviation or number (percent).

BMI, body mass index; WC, waist circumference; SBP, systolic blood pressure; DBP, diastolic blood pressure; FPG, fasting plasma glucose; ALT, alanine aminotransferase; HDL-C, high-density lipoprotein-cholesterol; eGFR, estimated glomerular filtration rate.

Table 3 demonstrates the multiple logistic regression model for the relationship between SRCs and metabolic syndrome with adjustment for other covariates, including age, gender, obesity, creatinine, uric acid, alcohol consumption, smoking, and regular exercise. SRCs were associated with an increased risk of metabolic syndrome (model 1, OR: 1.18, 95% CI: 1.06–1.34, *p* = 0.007). We further explored the relationship between the number and size of SRCs and metabolic syndrome (models 2–4), and found that the associated risk of metabolic syndrome was significantly higher in those with an SRC number ≥2 (model 2, OR: 1.35, 95% CI: 1.08–1.68, *p* = 0.008) and size ≥2 cm (model 3, OR: 1.33, 95% CI: 1.10–1.61, *p* = 0.003). Model 4 revealed that when the number and size of the SRCs were considered concomitantly, those with an SRC size ≥2 cm had higher associated risk of metabolic syndrome, regardless of whether the number of SRCs was single (OR:1.30, 95% CI: 1.04–1.62, *p* = 0.021) or multiple (OR: 1.42, 95% CI: 1.02–1.98, *p* = 0.040). In subjects with an SRC number ≥2 and < 2 cm, the associated risk for metabolic syndrome was statistically borderline (OR: 1.30, 95% CI: 0.98–1.73, *p* = 0.065). Singular SRCs of < 2 cm were not associated with higher risk of metabolic syndrome (OR: 1.04, 95% CI: 0.88–1.24, *p* = 0.645). However, both number ≥2/size ≥2 cm (OR: 1.42, 95% CI: 1.02–1.98, *p* = 0.04) and number < 2/size ≥2 cm (OR: 1.30, 95% CI: 1.04–1.62, *p* = 0.021) were positively related to metabolic syndrome. In addition, metabolic syndrome was also positively associated with older age, obesity, uric acid level and smoking, but inversely related to regular exercise.

**Table 3:** Logistic regression models for the association of simple renal cysts with metabolic syndrome.

	**Model 1**	**Model 2**	**Model 3**	**Model 4**
	**OR (95% CI)**	**OR (95% CI)**	**OR (95% CI)**	**OR (95% CI)**
Age, 40–65 vs. < 40 years	2.84 (2.53–3.19)^***^	2.84 (2.53–3.19)^***^	2.84 (2.53–3.19)^***^	2.84 (2.53–3.19)^***^
Age, >65 vs. < 40 years	5.88 (5.06–6.84)^***^	5.84 (5.02–6.80)^***^	5.85 (5.03–6.81)^***^	5.82 (5.00–6.77)^***^
Male vs. female	0.94 (0.85–1.04)	0.94 (0.85–1.03)	0.94 (0.85–1.04)	0.94 (0.85–1.03)
Obesity, yes vs. no	5.92 (5.42–6.45)^***^	5.92 (5.42–6.46)^***^	5.91 (5.42–6.45)^***^	5.92 (5.42–6.46)^***^
Creatinine, mg/dl	0.91 (0.82–1.02)	0.91 (0.81–1.02)	0.91 (0.82–1.02)	0.91 (0.81–1.02)
Uric acid, mg/dl	1.31 (1.27–1.35)^***^	1.31 (1.27–1.35)^***^	1.31 (1.27–1.35)^***^	1.31 (1.27–1.35)^***^
Current alcohol consumption, yes vs. no	0.92 (0.81–1.04)	0.92 (0.80–1.04)	0.92 (0.81–1.04)	0.92 (0.80–1.04)
Current smoking, yes vs. no	1.50 (1.32–1.71)^***^	1.51 (1.32–1.71)^***^	1.50 (1.32–1.71)^***^	1.50 (1.32–1.71)^***^
Regular exercise ≥3/week, yes vs. no	0.75 (0.64–0.87)^***^	0.75 (0.65–0.88)^***^	0.75 (0.64–0.88)^***^	0.75 (0.65–0.88)^***^
Simple renal cysts, yes vs. no	**1.18 (1.05**–1.34)^**^			
**Number of simple renal cysts**				
<2 vs. none		1.13 (0.98–1.30)		
≥2 vs. none		1.35 (1.08–1.68)^**^		
**Size of simple renal cysts**				
<2 cm vs. none			1.10 (0.95–1.28)	
≥2 cm vs. none			1.33 (1.10–1.61)^**^	
**Combination of number and size**				
Number < 2 and size < 2 cm vs. none				1.04 (0.88–1.24)
Number ≥2 and size < 2 cm vs. none				1.30 (0.98–1.73)
Number < 2 and size ≥2 cm vs. none				1.30 (1.04–1.62)^*^
Number ≥2 and size ≥2 cm vs. none				1.42 (1.02–1.98)^*^

* p < 0.05.

** p < 0.01.

*** p < 0.001.

OR, odds ratio; CI, confidence interval.

Bold values denote statistical significance at the *p* < 0.05 level.

## Discussion

### Principal findings

Our study found that presence of SRCs is positively associated with risk of metabolic syndrome, especially in those with larger or plural SRCs. To the best of our knowledge, this is the first study to investigate the association of SRCs and its characteristics with metabolic syndrome with adjustments for many important cardio-metabolic variables in a relatively large population. Although the mechanism underlying the link between SRCs and metabolic syndrome is still unclear, recent studies showed that SRCs were found to be associated with an increased risk of albuminuria in young adults (24) and SRCs are also associated with worse renal function in type 2 diabetes patients (25). The negative impact of SRCs in general population of this study was similar to young adults and diabetes patients in the two studies mentioned above (24, 25).

### SRCs and metabolic disorders

According to previous studies about the association between SRCs and cardio-metabolic factors, it has been confirmed that SRCs are associated with elevated blood pressure (10, 26). Lee et al. found that SRCs were related to prehypertension and hypertension and larger (size ≥2 cm) and multiple renal cysts (number ≥2), which further elevated the associated risk of prehypertension and hypertension (10). Hong et al. (26) also demonstrated a positive relationship between hypertension and SRCs, especially in those with larger (size ≥1.4 cm) or multiple (number ≥2) SRCs (26). Nevertheless, the association between SRCs and DM remains inconsistent (4, 7–9, 11), and adjustments for important covariates, such as age (7), sex (7), blood pressure (7–9), and lifestyle habits (e.g. smoking (7–9, 11), alcohol consumption (4, 7–9, 11) and exercise (4, 7–9, 11) are lacking in previous studies. Although high BMI was positively associated with SRCs in a healthy Korean population (11), the association of SRCs with central obesity and dyslipidemia has not been studied. Our study showed that subjects with SRCs may have a higher associated risk of metabolic syndrome after adjusting other variables. The associated risk of metabolic syndrome were higher in subjects with SRCs, especially for sizes ≥2 cm or numbers ≥2.

### Mechanism of SRCs and metabolic syndrome

The exact mechanism for the relationship between SRCs and metabolic syndrome remains unclear. However, SRCs were found to have impacts on (1) renin-angiotensin-aldosterone system (RAAS), (2) insulin resistance, and (3) chronic inflammation, which are all essential in the pathogenesis of metabolic syndrome (27, 28). The serum renin level was higher in subjects with multiple (number ≥2) and larger (size ≥2 cm) SRCs (10), which may increase RAAS activity and contribute to the development of elevated blood pressure (29, 30). In addition, renalase, derived from the kidney, of which levels were lower in subjects with renal cysts (31), may play a role through its degradation of serum catecholamines in the blood (32). Lower renalase levels in subjects with SRCs (31) may possibly result in excess catecholamine, and thus partially contributes to increased insulin resistance. We also additionally analyzed the relationship between SRCs and MS with concomitant consideration of individual MS component (Supplementary Table 2). The statistical significance of the positive relationship between SRCs and MS were attenuated to be insignificant when elevated blood pressure or hyperglycemia was adjusted, but the association between SRCs and MS remained significant when hypertriglyceridemia, low HDL-C or central obesity was adjusted in separate models. The results provided that the association of SRCs and MS might be mediated by abnormal blood pressure and hyperglycemia. As for SRCs and chronic inflammation, subjects with SRCs had more activated RAAS, which may induce increased systemic inflammation (33, 34). Furthermore, excess of catecholamine caused by lower renalase levels in subjects with SRCs, also potentially increased systemic inflammation (35–37). Therefore, elevated activity of RAAS, increased insulin resistance and chronic systemic inflammation may help to explain the pathogenesis of metabolic syndrome in subjects with SRCs.

In this study, metabolic syndrome was also shown to be positively related to older age, obesity, uric acid level and cigarette smoking; in contrast, regular exercise was inversely related to the risk of metabolic syndrome. These results are compatible with previous studies (15, 38–40). Aging was commonly associated with increased body fat, decreased muscle mass (22) and increased oxidative stress (41), which were all crucial for developing insulin resistance and subsequent metabolic syndrome (22, 27, 42). In addition, age was also found to be associated with formation and growth of SRCs (43). Previous study also showed an increase in the frequency of cysts with advanced age and growth in volume with a doubling of their volume after about 10 years (6). Besides, our analysis also showed that age was the major factor that associated with SRCs (shown in Supplementary Table 1). As a result, age may potentially modulate the interaction between SRCs and MS. Obesity was associated with visceral fat deposit, elevated free fatty acid levels, and increased systemic inflammatory cytokines (27, 44), which may inevitably result in the evolution of metabolic syndrome. As for uric acid level and metabolic syndrome, previous studies have demonstrated that uric acid not only induced hepatic gluconeogenesis, hepatic fat accumulation and oxidative stress, but also exacerbated the development of insulin resistance, hypertension and metabolic syndrome (45, 46). Smoking was also found to be associated with metabolic syndrome through its effect on elevating blood pressure, increasing insulin resistance and altering lipid metabolism (40). A previous study found that exercise helped correct metabolic imbalance by reducing blood pressure, blood sugar and triglyceride levels, and waist circumference, as well as by increasing the serum HDL-C level (40), which may partially explain the inverse relationship between regular exercise and metabolic syndrome.

### Limitation

This study has the strength of a relatively large sample, comprehensive serologic data and concomitant adjustment of important covariates of metabolic syndrome. However, there are also several limitations to this study. First, because this study had a cross-sectional design, it is impossible to establish a causal relationship between metabolic syndrome and renal cysts. Second, we did not obtain the detailed dietary histories of the participants in this study. Third, the information of some diseases such as maturity onset diabetes of the young type 5 (MODY 5) or hyperaldosteronism, which may correlate renal cyst with metabolic syndrome (47–50), is lacking. Although the prevalence of MODY 5 and hyperaldosteronism is relatively low, further study with comprehensive information of such diseases is still helpful to clarify their impacts on relationship between SRCs and metabolic syndrome. Fourth, thorough evaluation of metabolic syndrome includes more specific laboratory information including high sensitivity C-reactive protein, inflammatory cytokines such as tumor necrosis factor-alpha, interleukin-6, homeostatic model assessment for insulin resistance, microalbuminuria as well as endothelial function measurements (51–54), which were not examined in this study. Apart from this, the serum renalase, an enzyme that may potentially explain the interaction between renal cysts and metabolic syndrome, was not available. Therefore, future investigation that takes account of both renalase and thorough evaluation of metabolic syndrome may be necessary to disclose the possible mechanism about the relationship between SRCs and metabolic syndrome.

In conclusion, SRCs were related to a higher associated risk of metabolic syndrome, and the association is more significant in those with larger (sizes ≥2 cm) or plural (numbers ≥2) SRCs. Our data suggest that presence of SRCs, even with incidental finding, might require a further survey of potential metabolic syndrome. Although there have been some predictive models of metabolic syndrome (55–57), clinical application of SRCs in the risk stratification needs further investigation.

## Data availability statement

The original contributions presented in the study are included in the article/Supplementary material, further inquiries can be directed to the corresponding author.

## Ethics statement

The studies involving human participants were reviewed and approved by the Institutional Review Board of National Cheng Kung University Hospital, Tainan, Taiwan. Written informed consent for participation was not required for this study in accordance with the national legislation and the institutional requirements.

## Author contributions

Conceptualization: W-CS. Methodology: W-CS, Z-JS, and C-YC. Formal analysis and investigation: Z-JS and C-YC. Writing—original draft preparation: W-CS and Y-TC. Writing—review and editing: W-CS, Y-TC, and J-SW. Resources: Y-TC, F-HL, Y-CY, C-JC, and J-SW. Supervision: J-SW. All authors contributed to the article and approved the submitted version.

## Funding

This research was funded by the National Cheng Kung University Hospital, Taiwan (Grant Number: NCKUH-11103034).

## Conflict of interest

The authors declare that the research was conducted in the absence of any commercial or financial relationships that could be construed as a potential conflict of interest.

## Publisher's note

All claims expressed in this article are solely those of the authors and do not necessarily represent those of their affiliated organizations, or those of the publisher, the editors and the reviewers. Any product that may be evaluated in this article, or claim that may be made by its manufacturer, is not guaranteed or endorsed by the publisher.

## References

[1] Katabathina VS Kota G Dasyam AK Shanbhogue AK Prasad SR. Adult renal cystic disease: a genetic, biological, and developmental primer. Radiographics. (2010) 30:1509–23. 10.1148/rg.306105513 21071372

[2] Bonsib SM. The classification of renal cystic diseases and other congenital malformations of the kidney and urinary tract. Arch Pathol Lab Med. (2010) 134:554–68. 10.5858/134.4.554 20367308

[3] Simms RJ Ong AC. How simple are 'simple renal cysts'? Nephrol Dial Transplant. (2014) 29:iv106–12. 10.1093/ndt/gfu106 25165175 PMC4158337

[4] Terada N Arai Y Kinukawa N Yoshimura K Terai A. Risk factors for renal cysts. BJU Int. (2004) 93:1300–2. 10.1111/j.1464-410X.2004.04844.x 15180627

[5] Pedersen JF Emamian SA Nielsen MB. Simple renal cyst: relations to age and arterial blood pressure. Br J Radiol. (1993) 66:581–4. 10.1259/0007-1285-66-787-581 8374720

[6] Chang CC Kuo JY Chan WL Chen KK Chang LS. Prevalence and clinical characteristics of simple renal cyst. J Chin Med Assoc. (2007) 70:486–91. 10.1016/S1726-4901(08)70046-7 18063502

[7] Chen J Ma X Xu D Cao W Kong X. Association between simple renal cyst and kidney damage in a Chinese cohort study. Ren Fail. (2019) 41:600–6. 10.1080/0886022X.2019.1632718 31282239 PMC6691781

[8] Han Y Zhang M Lu J Zhang L Han J Zhao F. Hyperuricemia and overexcretion of uric acid increase the risk of simple renal cysts in type 2 diabetes. Sci Rep. (2017) 7:3802. 10.1038/s41598-017-04036-6 28630500 PMC5476589

[9] Ozveren B Onganer E Turkeri LN. Simple renal cysts: prevalence, associated risk factors and follow-up in a health screening cohort. Urol J. (2016) 13:2569–75. 26945663

[10] Lee CT Yang YC Wu JS Chang YF Huang YH Lu FH. Multiple and large simple renal cysts are associated with prehypertension and hypertension. Kidney Int. (2013) 83:924–30. 10.1038/ki.2012.481 23389415

[11] Choi JD. Clinical characteristics and long-term observation of simple renal cysts in a healthy Korean population. Int Urol Nephrol. (2016) 48:319–24. 10.1007/s11255-015-1186-7 26685889

[12] Eckel RH Grundy SM Zimmet PZ. The metabolic syndrome. Lancet. (2005) 365:1415–28. 10.1016/S0140-6736(05)66378-7 15836891

[13] Kip KE Marroquin OC Kelley DE Johnson BD Kelsey SF Shaw LJ. Clinical importance of obesity versus the metabolic syndrome in cardiovascular risk in women: a report from the Women's Ischemia Syndrome Evaluation (WISE) study. Circulation. (2004) 109:706–13. 10.1161/01.CIR.0000115514.44135.A8 14970104

[14] Smith SR. Importance of diagnosing and treating the metabolic syndrome in reducing cardiovascular risk. Obesity. (2006) 14(Suppl 3):128S−34S. 10.1038/oby.2006.292 16931494

[15] Grundy SM Cleeman JI Daniels SR Donato KA Eckel RH Franklin BA Jr. Diagnosis and management of the metabolic syndrome: an American Heart Association/National Heart, Lung, and Blood Institute Scientific Statement. Circulation. (2005) 112:2735–52. 10.1161/CIRCULATIONAHA.105.169404 16157765

[16] Moreira GC Cipullo JP Ciorlia LA Cesarino CB Vilela-Martin JF. Prevalence of metabolic syndrome: association with risk factors and cardiovascular complications in an urban population. PLoS ONE. (2014) 9:e105056. 10.1371/journal.pone.0105056 25180496 PMC4152120

[17] Kong X Ma X Zhang C Su H Gong X Xu D. Increased risk of kidney damage among Chinese adults with simple renal cyst. Int Urol Nephrol. (2018) 50:1687–94. 10.1007/s11255-018-1880-3 29728991

[18] Hasegawa EM Fuller R Chammas MC de Mello FM Goldenstein-Schainberg C. Increased prevalence of simple renal cysts in patients with gout. Rheumatol Int. (2013) 33:413–6. 10.1007/s00296-012-2380-x 22453524

[19] Chou YT Li CH Shen WC Yang YC Lu FH Wu JS Chang CJ. Association of sleep quality and sleep duration with serum uric acid levels in adults. PLoS ONE. (2020) 15:e0239185. 10.1371/journal.pone.0239185 32941519 PMC7497980

[20] Chu NF. Prevalence of obesity in Taiwan. Obes Rev. (2005) 6:271–4. 10.1111/j.1467-789X.2005.00175.x 16246212

[21] American Diabetes A. Diagnosis and classification of diabetes mellitus. Diabetes Care. (2010) 33 Suppl 1:S62–9. 10.2337/dc10-S062 20042775 PMC2797383

[22] Alberti KG Zimmet P Shaw J. Metabolic syndrome–a new world-wide definition. A consensus statement from the International Diabetes Federation. Diabet Med. (2006) 23:469–80. 10.1111/j.1464-5491.2006.01858.x 16681555

[23] Whelan TF. Guidelines on the management of renal cyst disease. Can Urol Assoc J. (2010) 4:98–9. 10.5489/cuaj.10023 20368890 PMC2845761

[24] Boo HJ Lee JE Chung SM Jang HR Huh W Kim DJ. The presence of simple renal cysts is associated with an increased risk of albuminuria in young adults. Korean J Intern Med. (2022) 37:425–33. 10.3904/kjim.2020.576 34865415 PMC8925965

[25] Wei L Xiao Y Xiong X Li L Yang Y Han Y. The relationship between simple renal cysts and renal function in patients with type 2 diabetes. Front Physiol. (2020) 11:616167. 10.3389/fphys.2020.616167 33384617 PMC7770177

[26] Hong S Lim JH Jeong IG Choe J Kim CS Hong JH. What association exists between hypertension and simple renal cyst in a screened population? J Hum Hypertens. (2013) 27:539–44. 10.1038/jhh.2013.12 23466877

[27] Rochlani Y Pothineni NV Kovelamudi S Mehta JL. Metabolic syndrome: pathophysiology, management, and modulation by natural compounds. Ther Adv Cardiovasc Dis. (2017) 11:215–25. 10.1177/1753944717711379 28639538 PMC5933580

[28] Zhu L Zhao X Zeng P Zhu J Yang S Liu A. Study on autonomic dysfunction and metabolic syndrome in Chinese patients. J Diabetes Investig. (2016) 7:901–7. 10.1111/jdi.12524 27181217 PMC5089954

[29] Ribeiro-Oliveira A Jr. Nogueira AI Pereira RM Boas WW Dos Santos RA Simões e Silva AC. The renin-angiotensin system and diabetes: an update. Vasc Health Risk Manag. (2008) 4:787–803. 10.2147/VHRM.S1905 19065996 PMC2597759

[30] Das UN. Renin-angiotensin-aldosterone system in insulin resistance and metabolic syndrome. J Transl Intern Med. (2016) 4:66–72. 10.1515/jtim-2016-0022 28191524 PMC5290902

[31] Elcioglu OC Afsar B Takir M Toprak AE Bakan A Bakan S. Renalase: another puzzle piece between hypertension and simple renal cysts? Int Urol Nephrol. (2015) 47:1181–6. 10.1007/s11255-015-1008-y 25987344

[32] Luft FC. Renalase, a catecholamine-metabolizing hormone from the kidney. Cell Metab. (2005) 1:358–60. 10.1016/j.cmet.2005.05.008 16054084

[33] Crowley SD Rudemiller NP. Immunologic effects of the renin-angiotensin system. J Am Soc Nephrol. (2017) 28:1350–61. 10.1681/ASN.2016101066 28151411 PMC5407736

[34] Sriramula S Francis J. Tumor necrosis factor - alpha is essential for angiotensin ii-induced ventricular remodeling: role for oxidative stress. PLoS ONE. (2015) 10:e0138372. 10.1371/journal.pone.0138372 26378790 PMC4574701

[35] Li G Xu J Wang P Velazquez H Li Y Wu Y. Catecholamines regulate the activity, secretion, and synthesis of renalase. Circulation. (2008) 117:1277–82. 10.1161/CIRCULATIONAHA.107.732032 18299506

[36] Guo X Wang L Velazquez H Safirstein R Desir GV. Renalase: its role as a cytokine, and an update on its association with type 1 diabetes and ischemic stroke. Curr Opin Nephrol Hypertens. (2014) 23:513–8. 10.1097/MNH.0000000000000044 24992568 PMC4383282

[37] Flierl MA Rittirsch D Huber-Lang M Sarma JV Ward PA. Catecholamines-crafty weapons in the inflammatory arsenal of immune/inflammatory cells or opening pandora's box? Mol Med. (2008) 14:195–204. 10.2119/2007-00105.Flierl 18079995 PMC2136428

[38] Saklayen MG. The global epidemic of the metabolic syndrome. Curr Hypertens Rep. (2018) 20:12. 10.1007/s11906-018-0812-z 29480368 PMC5866840

[39] Carnethon MR Loria CM Hill JO Sidney S Savage PJ Liu K. Risk factors for the metabolic syndrome: the coronary artery risk development in young adults (CARDIA) study, 1985-2001. Diabetes Care. (2004) 27:2707–15. 10.2337/diacare.27.11.2707 15505009

[40] Sun K Liu J Ning G. Active smoking and risk of metabolic syndrome: a meta-analysis of prospective studies. PLoS ONE. (2012) 7:e47791. 10.1371/journal.pone.0047791 23082217 PMC3474781

[41] El Assar M Angulo J Rodríguez-Mañas L. Oxidative stress and vascular inflammation in aging. Free Radic Biol Med. (2013) 65:380–401. 10.1016/j.freeradbiomed.2013.07.003 23851032

[42] Hildrum B Mykletun A Hole T Midthjell K Dahl AA. Age-specific prevalence of the metabolic syndrome defined by the International Diabetes Federation and the National Cholesterol Education Program: the Norwegian HUNT 2 study. BMC Public Health. (2007) 7:220. 10.1186/1471-2458-7-220 17727697 PMC2048947

[43] Terada N Arai Y Kinukawa N Terai A. The 10-year natural history of simple renal cysts. Urology. (2008) 71:7–11. discussion 11–2. 10.1016/j.urology.2007.07.075 18242354

[44] Esser N Legrand-Poels S Piette J Scheen AJ Paquot N. Inflammation as a link between obesity, metabolic syndrome and type 2 diabetes. Diabetes Res Clin Pract. (2014) 105:141–50. 10.1016/j.diabres.2014.04.006 24798950

[45] Battelli MG Bortolotti M Polito L Bolognesi A. The role of xanthine oxidoreductase and uric acid in metabolic syndrome. Biochim Biophys Acta Mol Basis Dis. (2018) 1864:2557–65. 10.1016/j.bbadis.2018.05.003 29733945

[46] Kanbay M Jensen T Solak Y Le M Roncal-Jimenez C Rivard C. Uric acid in metabolic syndrome: from an innocent bystander to a central player. Eur J Intern Med. (2016) 29:3–8. 10.1016/j.ejim.2015.11.026 26703429 PMC4826346

[47] Calhoun DA. Aldosteronism and hypertension. Clin J Am Soc Nephrol. (2006) 1:1039–45. 10.2215/CJN.01060306 17699324

[48] Fallo F Pilon C Urbanet R. Primary aldosteronism and metabolic syndrome. Horm Metab Res. (2012) 44:208–14. 10.1055/s-0031-1295412 22116746

[49] Mateus JC Rivera C O'Meara M Valenzuela A Lizcano F. Maturity-onset diabetes of the young type 5 a multisystemic disease: a case report of a novel mutation in the HNF1B gene and literature review. Clin Diabetes Endocrinol. (2020) 6:16. 10.1186/s40842-020-00103-6 32864159 PMC7448977

[50] Shields BM Hicks S Shepherd MH Colclough K Hattersley AT Ellard S. Maturity-onset diabetes of the young (MODY): how many cases are we missing? Diabetologia. (2010) 53:2504–8. 10.1007/s00125-010-1799-4 20499044

[51] Mohammadi M Gozashti MH Aghadavood M Mehdizadeh MR Hayatbakhsh MM. Clinical significance of serum IL-6 and TNF-α levels in patients with metabolic syndrome. Rep Biochem Mol Biol. (2017) 6:74–9. 29090232 PMC5643447

[52] Singh Y Garg MK Tandon N Marwaha RK A. study of insulin resistance by HOMA-IR and its cut-off value to identify metabolic syndrome in urban Indian adolescents. J Clin Res Pediatr Endocrinol. (2013) 5:245–51. 10.4274/Jcrpe.1127 24379034 PMC3890224

[53] Tziomalos K Athyros VG Karagiannis A Mikhailidis DP. Endothelial dysfunction in metabolic syndrome: prevalence, pathogenesis and management. Nutr Metab Cardiovasc Dis. (2010) 20:140–6. 10.1016/j.numecd.2009.08.006 19833491

[54] Ridker PM Wilson PW Grundy SM. Should C-reactive protein be added to metabolic syndrome and to assessment of global cardiovascular risk? Circulation. (2004) 109:2818–25. 10.1161/01.CIR.0000132467.45278.59 15197153

[55] Guo H Jiang W Zhao B Xiong Y Lu Z A. Predictive model of metabolic syndrome by medical examination: evidence from an 8-year chinese cohort. Diabetes Metab Syndr Obes. (2021) 14:4459–67. 10.2147/DMSO.S314550 34795493 PMC8593343

[56] Soldatovic I Vukovic R Culafic D Gajic M Dimitrijevic-Sreckovic V. siMS score: simple method for quantifying metabolic syndrome. PLoS ONE. (2016) 11:e0146143. 10.1371/journal.pone.0146143 26745635 PMC4706421

[57] Kim J Mun S Lee S Jeong K Baek Y. Prediction of metabolic and pre-metabolic syndromes using machine learning models with anthropometric, lifestyle, and biochemical factors from a middle-aged population in Korea. BMC Public Health. (2022) 22:664. 10.1186/s12889-022-13131-x 35387629 PMC8985311

