# Supplementary material for: Association of simple renal cysts with metabolic syndrome in adults
Source: Front Public Health. 2022 Nov 3;10:951638. doi: 10.3389/fpubh.2022.951638 (PMC9669605; doi:10.3389/fpubh.2022.951638)
Supplement: Supplementary file 1 [file Table_1.docx]

**Supplement table 1.** Logistic regression model for renal cyst with age

| Variables | Adjusted OR  (95% CI) | P value |
| --- | --- | --- |
| Age, 40-65 yrs vs <40yrs | 3.45 (2.79-4.28) | <0.001 |
| Age, > 65 yrs vs <40yrs | 8.15 (6.34-10.47) | <0.001 |
| Male vs female | 2.06 (1.83-2.32) | <0.001 |
| eGFR, mL/min/1.73m^2^ | 0.99 (0.99-0.99) | <0.001 |
| Hypertension, yes vs no | 1.45 (1.06-2.33) | <0.001 |
| OR: odds ratio, CI: confidence interval | | |

**Supplement table 2**. The relationship between simple renal cysts (predictor variable) and metabolic syndrome (outcome variable) with consideration of individual component of metabolic syndrome

|  | Model 1^a^ | Model 2^a^ | Model 3^a^ | Model 4^a^ | Model 5^a^ |  |
| --- | --- | --- | --- | --- | --- | --- |
|  | OR (95% CI) | OR (95% CI) | OR (95% CI) | OR (95% CI) | OR (95% CI) |  |
| Simple renal cysts, yes vs no | 1.02  (0.89 -1.17) | 1.12  (0.98 -1.29) | 1.24  (1.07 -1.43)^**^ | 1.28  (1.12 -1.47)^***^ | 1.16  (1.02 -1.33)^*^ |  |
| Elevated blood pressure^b^, yes | 7.26  (6.62-7.95)^***^ |  |  |  |  |  |
| Hyperglycemia^c^, yes |  | 12.74  (11.52-14.08)^***^ |  |  |  |  |
| Hypertriglyceridemia^d^, yes |  |  | 16.30  (14.72-18.05)^***^ |  |  |  |
| Low HDL-C^e^, yes |  |  |  | 10.03  (9.12-11.03)^***^ |  |  |
| Central obesity^f^, yes |  |  |  |  | 11.99  (10.76-13.36)^***^ |  |
| HDL-C, high-density lipoprotein-cholesterol  ^a^All models were adjusted for age, sex, obesity, creatinine, uric acid, current smoking, current alcohol drinking and exercise ^b^elevated blood pressure: blood pressure ≥ 130/85 mmHg or under treatment of previously diagnosed hypertension , ^c^hyperglycemia: fasting plasma glucose ≥ 100 mg/dl or previously diagnosed type 2 diabetes, ^d^hypertriglyceridemia: triglyceride ≥ 150 mg/dl or under drug treatment for dyslipidemia, ^e^low HDL-C: HDL-C <40 mg/dL in males and <50 in females, ^f^central obesity: waist circumference ≥ 90 cm in men or ≥ 80 cm in women,  ^*^ p < 0.05, ^**^ p < 0.01, ^***^ p < 0.001. | | | | | |  |
